# THE THINGS WE KEEP: AN EXAMINATION INTO THE USE OF MATERIAL POSSESSIONS IN QUALITATIVE GERONTOLOGICAL INTERVIEWS

**DOI:** 10.1093/geroni/igad104.1946

**Published:** 2023-12-21

**Authors:** Samuel Van Vleet

**Affiliations:** Miami University, Oxford, Ohio, United States

## Abstract

Qualitative interviews are a useful research method because they allow researchers to gather in-depth data beyond the use of traditional survey methods. In this way, qualitative research is an effective method to understand complex and intricate aging-related concepts because there is room for developing new methodological ways for gathering data. A possible way to expand data collections is through the incorporation of material possessions as research aids. Older adults likely accumulate a series of material objects throughout their life course. These objects can take on a supportive role for older adults by conveying important aspects of meaning and are often attached to their identity. Some items have direct associations with later life changes (canes, glasses, etc) while other items (photographs and mementos) are linked with an individual’s identity and accrue a sense of meaning. While previous researchers have documented the importance of objects in later life through material convoys, little has been done to incorporate these meaningful objects into qualitative interview methodology. This paper assesses how incorporations of material possessions into qualitative interviews can assist researchers in gathering rich data. Through a structured literature review, this paper highlights the key findings, past incorporations, and benefits for qualitative interview methodology. Findings suggest that positive outcomes and enriched data result from incorporating material possessions as aides to qualitative interviews. Future researchers should consider how their studies can benefit from the incorporation of meaningful objects in later life.

